# The Economic Burden of Dementia: Evidence from a Survey of Households of People with Dementia and Their Caregivers

**DOI:** 10.3390/ijerph18052717

**Published:** 2021-03-08

**Authors:** Hikaru Oba, Yoshihiko Kadoya, Haruka Okamoto, Teruyuki Matsuoka, Yoshinari Abe, Keisuke Shibata, Jin Narumoto

**Affiliations:** 1 Department of Psychiatry, Graduate School of Medical Science, Kyoto Prefectural University of Medicine, Kyoto 602-8566, Japan; ha-okamo@koto.kpu-m.ac.jp (H.O.); tmms2004@koto.kpu-m.ac.jp (T.M.); abeyoshi@koto.kpu-m.ac.jp (Y.A.); kei-siv@koto.kpu-m.ac.jp (K.S.); jnaru@koto.kpu-m.ac.jp (J.N.); 2 Graduate School of Human Sciences, Osaka University, Osaka 565-0871, Japan; 3 School of Economics, Hiroshima University, Hiroshima 739-8525, Japan; ykadoya@hiroshima-u.ac.jp

**Keywords:** adult guardianship, civil trust system, financial management, financial capacity, family caregiver

## Abstract

Although a cognitive impairment such as dementia causes serious economic problems among older people, its impact on economic activities is unclear. This study investigated the actual conditions of economic activities and the current status of the financial support systems among people with dementia and caregivers. One hundred and five dyads participated in the survey. Each dyad consisted of an older person with Alzheimer’s disease and their caregiver. The Mini-Mental State Examination (MMSE) and Functional Assessment Staging (FAST) were used to evaluate the cognitive functions of people with dementia. The caregivers were asked questions concerning the financial status of the household and their utilization of the financial support systems available to people with dementia. Average monthly care costs significantly increased according to the severity of dementia, while household income and spending incurred no significant changes. People with dementia experienced financial problems (including a large amount of erroneously purchased, unnecessary shopping), even though their assets were informally managed by their caregivers. Financial support systems such as adult guardianship and civil trust systems were rarely known and used. We proposed the propagation of the adult guardianship and civil trust systems and the development of contract guidelines for elderly customers including people with dementia.

## 1. Introduction

Various cognitive dysfunctions including memory, orientation, and executive functioning make it difficult for people with dementia to undertake economic activities. For instance, people with dementia sometimes repeatedly purchase the same product and are unable to calculate the change due to memory impairment [1]. Other studies have shown that cognitive dysfunction was associated with greater risk aversion related to poor financial decision-making and investments such as stockholding [2,3,4,5]. Older people with cognitive decline reduce household spending even if they do not have dementia [6]. The Alzheimer’s Society [7] reported that many people with dementia experience difficulties or need some assistance when using financial institutions, which likewise indicates the necessity of providing support for them to pay bills or to use automatic teller machines (ATMs). These previous studies have suggested that people with dementia could have a higher risk regarding financial situations, including poverty (by the locking up of their assets) or exploitation (via fraud and financial abuse).

Adult guardianship and/or civil trust systems are useful for protecting the assets of people with dementia. In the Japanese adult guardianship system, for those having difficulties in managing their assets, the court entrusts an attorney to their family members or professions (including lawyers and social workers). Previously, the guardians have been expected to support incapacitated people through the management of their assets and the signing of contracts, such as Long-term Care Insurance services. This system emphasizes respecting the rights of incapacitated people based on the empowerment and normalization [8]. The civil trust system is used mainly among the family members and aims to entrust the assets of people who have anxieties regarding their financial management capacities to a named third person, i.e., a son or daughter without commercial gain. This system enables people with dementia and their caregivers to utilize their assets more flexibly and respectfully than the adult guardianship system. Older people living alone, which, as a phenomenon, has been increasing in recent years [9], were expected to actively use these systems because financial risks rise in the case of dementia. Although these financial support systems have many advantages to protect or utilize the assets of people with dementia, people rarely know of their existence and thus rarely use them. A recent elderly household survey conducted nationwide in Japan also showed that only 26.4% of people aged 65 years and over had knowledge of the adult guardianship system while 14.5% understood the civil trust system [10]. This result suggests that the assets of people with dementia are informally managed by their caregivers, a form of management that is not statutorily protected in many cases.

Dementia is the cause of a critical situation which can lead to increased household spending or decreased household income because people with dementia may have to use care services such as daycare or home-visit care, and their relatives are also required to provide care. A Japanese study investigating the economic opportunity loss among people with dementia and caregivers indicated that between 1 and 5 million Japanese Yen (JPY) (10,000 United States Dollar (USD) and 50,000 USD, respectively, at an exchange rate of 1 USD/100 JPY) were lost due to dementia because caregivers had to quit their jobs or decrease their work hours to care for people with dementia [11]. A survey conducted by the Ministry of Health, Labour and Welfare of Japan showed that the average monthly household spending and income for those aged 65 and above among nonworkers (the mean number of the household persons was 1.83) was 225,470 JPY and 185,413 JPY (2255 USD and 1854 USD, at 1 USD/100 JPY), respectively [12]. However, this survey did not exclusively target people with dementia or their caregivers, and so their economic activities remain unclear.

Building on previous literature, the current study aims to investigate the economic burden of dementia; that is, it investigates how dementia influences household spending and the income of people with dementia and their caregivers, how the assets of people with dementia are managed, the kinds of financial problems people with dementia experience, and the financial support systems recognized by caregivers. Since Japan has the highest proportion of elderly people (aged 60 and above) in the world [13], the findings will be useful for constructing and instituting a practical policy in a country with such a high elderly proportion.

## 2. Materials & Methods

### 2.1. Procedure

The survey was conducted from March 2017 to February 2019 at five hospitals in four cities in the Kansai region, Japan. Of these, four were general public hospitals (the number of hospital beds is between 153 and 1065) and one was the proprietary psychiatric hospital (the number of hospital beds is 448). The ratios of those aged 65 or above within the population in these cities in 2017 were between 27.5% and 36.8% (mean rate = 31.5%) while those of the entire nation of Japan was 27.7%. Participants were patients with Alzheimer’s disease and their caregivers who have continued to visit these hospitals and constructed favorable therapeutic relationships with the physicians; participants were required to provide personal information such as household income and spending. Primary physicians confirmed medical charts and excluded individuals who had severe physical or psychiatric symptoms that would hinder their participation in the survey. The diagnosis of Alzheimer’s disease was based on the National Institute of Neurological and Communicative Disease and Stroke-Alzheimer’s Disease and Related Disorders Association criteria [14].

Primary physicians asked patients and caregivers whether they had time to listen the explanation about the survey after the consultation. A researcher informed them of the contents of the survey after which the participants agreed to listen to the survey explanation. Then, those who consented to the survey took the questionnaire home since most participants did not remember the information on their household spending. When participants visited the hospital on another day, a researcher confirmed the answers on the questionnaires written by the caregivers in their homes through face-to-face interviews, while another researcher (a clinical psychologist) conducted the cognitive test with the people with dementia. Participants were allowed to refuse to answer if they did not want to. The sample’s size was set as referred by the previous Japanese study (*n* = 105) [11]. We had already recruited 162 older people (and their caregivers) who had visited the hospital in the research duration. Of these, 109 dyads (67.3%) of people with dementia and their family caregivers consented to participate in the survey. The ethics committee of the Kyoto Prefectural University of Medicine approved the study (Number: ERB-C-760-2).

### 2.2. Measures

#### 2.2.1. Sociodemographic Variables

Age and gender of both people with dementia and caregivers were collected. We also asked the caregivers about care length, the duration from the time caregivers began to be involved with care, as well as their relationship with the people with dementia (spouse, son, daughter, or other), utilization of the Long-term Care Insurance services for people with dementia, number of persons including noncaregivers in the household, and living status of people with dementia (living alone or not). The household’s financial status, represented by spending and income, was asked about as well. “Shared household finances” describes all situations wherein caregivers and people with dementia shared their household spending and income, even if they did not reside together. Average monthly household spending (sum of house rent, utility costs, medical or care costs, food budget, traffic expenses, costs of entertainment, refund to the debt, and others), income and care costs of the household, and income of people with dementia were also asked from the caregivers to obtain more comprehensive economic information.

With regard to the Long-term Care Insurance system in Japan, older people who were accredited as requiring care support from the public agency were divided into seven care levels with the primary difference being in the upper limit of monthly costs (50,030 JPY for care level 1 and 360,650 JPY for care level 7) covered by the public budget. In this system, the utilization price of the public care service (except for the costs such as meals and materials used for recreation and those paid by out-of-pocket spending) was defined by the government, and 70% to 90% of utility costs are paid by public budgets according to the annual income level of the users. If the cost used by older people is over the upper limit of the public norm, they must pay all costs that are over the limit. Thus, we asked about out-of-pocket spending regarding care costs for the participants.

Financial assistance is required when people with dementia find it difficult to manage financial matters independently. If that is the case, we asked those who assist people with dementia’s financial matters, the caregiver in most cases, for the reasons they provide such assistance. The choices we presented were that the people with dementia (a) had asked them, (b) showed noticeable difficulty in managing financial matters independently because of his/her cognitive decline, (c) because of his/her health issues other than dementia, and (d) because of a physical disability (e.g., broken bone). Financial problems were also inquired into, and the options were defined as follows: unfair contracts (e.g., contracts for expensive things from home visit salespersons or suggestions for unnecessary renovations), large amounts of unnecessary shopping (e.g., repeated purchase of the same thing), wastefulness (e.g., purchase of expensive things or meeting charitable donations that would not be considered ordinary), exploitation by a third person (e.g., lending money to a third person who announces themselves as a friend), and others. These options were set on the basis of the results of the pilot survey and the practical experience of some professionals whose specialties were psychiatry, psychology, or economics. The levels of knowledge (understanding as to the extent of explaining for others, having heard but being unable to explain, and not knowing at all) and utilization (under utilization, under consideration for use, and never been considered for use) of the adult guardianship and civil trust systems were asked about in relation to investigating the actual conditions of economic activities of people with dementia.

#### 2.2.2. Functional and Cognitive Assessment of People with Dementia

The Physical Self-Maintenance Scale (PSMS) [15] and the Tokyo Metropolitan Institute of Gerontology Index of Competence (TMIG-IC) [16] were employed to evaluate the basic and instrumental activities of daily living (BADL and IADL). PSMS evaluates the BADL through six items: toilet, feeding, dressing, grooming, physical ambulation, and bathing. TMIG-IC consists of 13 items regarding IADL, including using public transportation and preparing a meal. Both scales were binomially evaluated, a higher score indicating better IADL function. The Mini-Mental State Examination (MMSE) [17] was evaluated by the clinical psychologists to assess global cognitive functioning. The MMSE was comprised of orientation, registration, attention and calculation, recall, naming, repetition, 3-stage command, reading, writing, and copying. The score range was 0–30, a lower score indicating severe cognitive dysfunction. Functional Assessment Staging (FAST) [18] was evaluated by psychiatrists to assess the severity of dementia. FAST is set at seven stages from normal adult (stage 1) to severe Alzheimer’s dementia (stage 7), which can evaluate the integrated capacity to undertake daily living.

### 2.3. Statistical Analysis

Descriptive statistics of each variable were calculated according to household status. Wilcoxon rank sum tests and Kruskal–Wallis tests were performed to compare household spending, income, and care costs. Although household status (shared or not shared) was considered in the analysis, we could not exempt the effect of the number of persons in the household and their direct impact on household spending because common spending in each household according to individual persons (e.g., total utility costs vs. individual contributions thereof) could not be distinguished. Furthermore, during the collection of information on the income of people with dementia, we also included this in the analysis. Chi-square tests and Fisher’s exact tests were conducted to compare the experience of financial problems by household status and the differences in financial management by the severity of dementia. All statistical analyses were performed using R version 3.3.1 (R Core Team, Vienna, Austria) [19].

## 3. Results

The participant who was evaluated as FAST stage 2 (*n* = 1) showed very mild cognitive decline [18], and those who were already institutionalized (*n* = 3) were excluded from the analysis. A total of 105 dyads of older people with Alzheimer’s disease and their caregivers living in the community participated in the study. The descriptive statistics are shown in Table 1. Half of caregivers were the spouse of people with dementia (*n* = 50, 47.6%), or caregivers were the son or daughter (*n* = 46, 43.8%). The mean number of households was 2.56 ± 0.9 persons. Some people with dementia (*n* = 14, 13.6%) lived alone. Most of the caregivers (*n* = 76, 72.4%), including all spouses, were of the shared household with people with dementia, and average care length was 3.50 ± 2.8 years. Three-quarters of people with dementia (*n* = 80, 76.2%) showed FAST stage 4 or 5, which indicated a mild-to-moderate severity of dementia. More than half the people with dementia (*n* = 68, 64.8%) used the Long-term Care Insurance services, such as daycare centers and home visit care services.

Table 2 shows the financial status by the household status and by the severity of dementia. The median of average monthly household spending and income was 195,000 JPY and 300,000 JPY, respectively, which showed no significant difference relative to the severity of dementia (*χ*^2^ (2) = 3.93, *p* = 0.140 for spending; *χ*^2^ (2) = 0.487, *p* = 0.783 for income, respectively). Greater care costs were required when people with dementia had more severe dementia (*χ*^2^ (2) = 16.6, *p* < 0.001), and this tendency was notable in the case of the shared household being considered.

However, irrespective of the severity of dementia, care costs were no different in the case of patients and caregivers not sharing their households (*χ*^2^ (2) = 0.75, *p* = 0.686).

Of the monthly shared household income, 90,000 JPY (median, range 10,000 JPY–259,000 JPY) were included as the income of people with dementia, which was differentiated according to the gender (male was 140,000 JPY, female was 72,000 JPY, *w* = 850, *p* = 0.002). No significant difference was shown in the severity of dementia (*χ*^2^ (2) = 1.43, *p* = 0.490).

One-third of people with dementia (*n* = 31, 29.5%) experienced financial problems because of dementia. Those who lived alone experienced more frequent financial problems than those who did not, although it was not significant (50.0% vs. 25.8%, *χ*^2^ (1) = 2.34, *p* = 0.125). Table 3 shows the contents of financial problems, which indicates that the most frequent problem was a large amount of unnecessary shopping (*n* = 18, 58.1%), followed by unfair contract (*n* = 3, 9.7%).

Assets of people with dementia were managed by the caregiver (*n* = 73, 70.2%, including two guardians of people with dementia) in many cases, followed by both the people with dementia and their caregiver (*n* = 16, 15.4%) and people with dementia themselves (*n* = 15, 14.4%), which demonstrated a significant difference among FAST levels (Fisher’s exact test, *p* = 0.015) (Table 4). In 64.0% (*n* = 57) of cases, the caregiver began to manage the assets of people with dementia when the people with dementia began to experience serious difficulties in independently managing the assets. In some cases (*n* = 23, 25.8%), people with dementia requested the caregiver to manage their assets.

Some caregivers (*n* = 20, 19.0%) had known about the adult guardianship system, but utilization of it was little overall. Only 2% (*n* = 2) of caregivers used the adult guardianship system. The civil trust system (*n* = 5, 4.8%) was less widespread in use than the adult guardianship system; in short, no participants used it (Table 5).

## 4. Discussion

With respect to the severity of dementia, care costs increased while average monthly household spending and income were not significantly impacted. Some participants experienced financial problems such as a large amount of unnecessary shopping and unfair contracts, and here, the assets of people with dementia were informally managed by their caregivers in many cases without using the adult guardianship or civil trust systems. This would suggest that this scheme requires more promotion among the general public. The mean care length of caregivers was 3.5 ± 2.8 years, and most people with dementia were evaluated as being at FAST stage 4 or 5 indicating mild-to-moderate severity of dementia. Therefore, the present findings might reveal the financial activities in the early stages of those with dementia.

The new findings showed larger incomes and less spending than that in the national survey [12]; however, these might be affected by the difference in the sample size, i.e., the number of household persons (1.83 persons/household in the report vs. 2.56 persons/household in the present finding) from each household as well as the difference in the age of workers. Moreover, the income of people with dementia was differentiated by gender. Many women with dementia received a pension for the non- or independent worker, which is less than that for the salaried worker. The present findings suggest that women with dementia who have lost their spouses may require more financial support than men with dementia in the future.

These results also showed that household spending and income did not change, although the care cost rose in line with the severity of dementia, which indicates dementia’s effect on the households. The finding that care costs rose in parallel with the severity of dementia was consistent with the previous report [20]. Our finding also suggested that participants might manage increasing care costs by saving on another household-spending dimension such as entertainment. Given that the Japanese Long-term Care Insurance system is universally available to all elderly people in accordance with his/her care-needs level, the system may partly serve as a buffer for the potential increase in care costs. The findings also showed that caregivers rarely cover the care costs when they are from a different household relative to the people with dementia in question. Caregivers may not feel impelled to provide financial support for people with dementia because the income exceeded the spending in each household status. This also means that many households in the present study had economic allowances, which could not entirely be accounted for.

Cognitive dysfunction leads to abnormal economic activities [3,4,5,21]; in particular, people with dementia who live alone might have a higher risk of experiencing financial problems in comparison to those who do not. Large amounts of unnecessary shopping were the most frequent financial problem experienced by the participants. Moreover, 10% of participants experienced being led into an unfair contractual obligation. Our findings suggested that people with dementia whose caregivers have difficulty supervising them frequently have a higher risk of losing their assets. Most caregivers managed assets of people with dementia, given the difficulty to independently control their assets because of dementia. People with dementia in the present study did not experience exploitation from a third person, which might be influenced by the fact that caregivers managed their assets in many cases.

While the adult guardianship and civil trust systems that are in place to protect the assets of incapacitated people were rarely used, even these systems were known by caregivers. The low percentage of knowledge (19.0%) and utilization (2.0%) of the adult guardianship system was similar to that of the previous reports [10]. Procedures to apply the adult guardianship system are complicated, and the required application costs are expensive. Most caregivers might not feel the necessity of using these systems since they manage the people with dementia’s assets for them. However, previous studies showed that cognitive and functional impairment are strong and that financial dependence is a potential risk factor of elder abuse [22]. The lack of knowledge and utilization of these systems caused the informal management of assets of people with dementia by caregivers, which may include the potential risk of financial exploitation or abuse by using their assets privately and not for the benefit of the people with dementia. Further studies are required to detect the factors associated with the knowledge and utilization of the adult guardianship and civil trust systems.

There are several limitations to the present study. First, although we revealed the income of people with dementia, the household spending in the present study could not eliminate the effect on the number of persons within a particular household since it is affected by various factors such as common spending (e.g., utility charges) and the proportion of income and spending among each household person. Second, this study had a small sample size and survey items included the invasive questions such as household spending and income, which indicate that the representation could be biased. For example, detailed comparisons such as gender difference of patients and caregivers was difficult and participants who sought help might have exaggerated their answer. Interpretation of the present findings should be cautious. However, the accuracy of the diagnosis relative to the severity of dementia was strengthened because these factors were evaluated by a psychiatrist or a clinical psychologist who has professional knowledge and skills regarding dementia. Caregivers provided answers regarding the household information, not only via self-questionnaire but also via face-to-face interviews. These procedures could contribute to improving the reliability of the information. Third, the present study was not based on random sampling, and all participants visited the hospital; that is, they have already connected to at least one support resource of some kind, which may include some biases. Since older people who are socially isolated and have a greater risk of financial problems (including fraud and exploitation by a third person) [23] did not participate in the study, the results regarding financial problems might be underestimated. Fourth, the present study did not focus on people with dementia living in care facilities. Moreover, the findings were applicable only to patients with Alzheimer’s disease and their caregivers. Different characteristics of neuropsychiatric symptoms are shown by dementia subtypes [24,25], which may influence the economic activities of people with dementia and their caregivers. Further studies are necessary to reveal the actual condition, and a more comprehensive picture, of economic activities among people with dementia and caregivers in a variety of contexts. Despite these limitations, this study revealed the details of the status of economic activities, including household spending among people with dementia and their caregivers. Because Japan has the highest proportion of elderly citizens in the world, our findings provide insight to other countries where there have been increasing numbers of older people, including people with dementia.

## 5. Conclusions

The shrinking of other spending caused by rising care costs may threaten the life of both people with dementia and caregivers. Moreover, many people with dementia have prevented their autonomous economic activities from being managed, and their assets are informally managed by caregivers without using the form power of attorney such as by the adult guardianship and civil trust systems. Since the number of older people who live alone is increasing [9], protecting older people from financial exploitation (including fraud when they suffer from dementia) is important. However, ensuring that even those with dementia maintain a right to autonomous economic activities is also required. We propose that policymakers should seek to deliberately spread knowledge and thus the uptake of the adult guardianship and civil trust systems and develop the contract guidelines focusing on protecting elderly customers, including people with dementia, from infringement of their rights.

## Figures and Tables

**Table 1:** Descriptive statistics of participants.

Characteristics	Caregiver			People with Dementia		
	*n*	*%*	Min–Max	*n*	*%*	Min–Max
Gender (female)	63	60		76	72.4	
Age (years) ^1^	66.4 ± 11.6		47–88	80.8 ± 6.5		64–94
Care length (years)	3.50 ± 2.8		0–15			
Relationship with people with dementia						
Spouse	50	47.6				
Son or daughter	46	43.8				
Other	9	8.6				
Household status (shared)	76	72.4				
Number of persons in household	2.56 ± 0.9		1–6			
Living alone (*n* = 103)				14	13.6	
Using LCIS				68	64.8	
Care level						
No accredited				32	30.5	
3				36	34.3	
4				20	19.0	
≥5				17	16.2	
PSMS ^1^				3.37 ± 2.1		0–6
TMIG-IC ^1^				4.86 ± 3.8		0–13
MMSE ^1^				16.4 ± 5.8		0–28
FAST						
4 (mild)				44	41.9	
5 (moderate)				36	34.3	
≥6 (severe)				25	23.8	

Note. *n* = 105. ^1^ Mean ± SD. SD, Standard Deviation; LCIS, Long-term Care Insurance Services; PSMS; Physical Self-Maintenance Scale; TMIG-IC, Tokyo Metropolitan Institute of Gerontology Index of Competence; MMSE, Mini-Mental State Examination; FAST, Functional Assessment Staging.

**Table 2:** Monthly average of spending, income, and care costs in each household.

Household	FAST	Total				Household Status							
						Shared				Not Shared			
		*n*	Median	Percentile (25%, 75%)	*p*	*n*	Median	Percentile (25%, 75%)	*p*	*n*	Median	Percentile (25%, 75%)	*p*
Spending	All	87	195.0	160.0, 285.5	0.140	62	184.5	155.0, 231.3	0.212	25	290.0	185.0, 390.0	0.386
	4	37	182.0	155.0, 204.0		27	170.0	152.0, 200.0		10	241.5	166.8, 380.0	
	5	30	196.0	160.8, 269.3		20	190.0	157.5, 238.8		10	255.0	190.8, 333.3	
	≥6	20	251.5	173.3, 327.5		15	200.0	169.0, 306.5		5	305.0	300.0, 500.0	
Income	All	94	300.0	216.3, 450.0	0.783	69	280.0	200.0, 380.0	0.989	25	440.0	350.0, 560.0	0.828
	4	42	282.0	211.5, 455.0		31	270.0	213.0, 385.0		11	440.0	225.0, 880.0	
	5	31	340.0	215.0, 475.0		22	288.5	196.3, 364.3		9	400.0	367.0, 560.0	
	≥6	21	300.0	250.0, 450.0		16	275.0	210.0, 382.5		5	450.0	400.0, 450.0	
Care costs	All	84	9.5	0, 20.0	<0.001	60	10.0	0, 25.0	<0.001	24	0	0, 10.0	0.686
	4	35	0	0, 10.0		26	0	0, 9.8		9	0	0, 10.0	
	5	29	10.0	0, 20.0		18	12.5	1.5, 20.0		11	0	0, 15.0	
	≥6	20	30.0	10.0, 40.0		16	32.0	26.5, 44.5		4	0	0, 2.5	

**Table 3:** Managing financial problems in dementia.

Financial Problems	*n*	*%*
Large amount of unnecessary shopping (e.g., repeatedly purchase of the same thing)	18	58.1
Unfair contracts (e.g., contracts for expensive things from home-visit salespersons or suggestions for unnecessary renovations)	3	9.7
Wastefulness (e.g., purchase of expensive things or meeting charitable donations that would not be considered ordinary)	2	6.5
Exploitation by a third person (e.g., lending money to a third person who announces themselves as a friend)	0	0
Others	7	22.6
Unknown	6	19.4

Note. *n* = 31, multiple answer.

**Table 4:** Managing finances for people with dementia.

Management	Total		FAST 4		FAST 5		FAST 6		*p*
	*n*	*%*	*n*	*%*	*n*	*%*	*n*	*%*	
Self	15	14.4	11	25.0	3	8.6	1	4.0	0.015
Caregiver	73	70.2	24	54.5	26	74.3	23	92.0	
Both	16	15.4	9	20.5	6	17.1	1	4.0	

Note. *n* = 104; two guardians (son or daughter of people with dementia) were included as caregivers. FAST, Functional Assessment Staging.

**Table 5:** Knowledge and utilization status of adult guardianship and civil trust systems.

Status	Adult Guardianship						Civil Trust System					
	Household Status						Household Status					
	Total		Shared		Not Shared		Total		Shared		Not Shared	
	*n*	*%*	*n*	*%*	*n*	*%*	*n*	*%*	*n*	*%*	*n*	*%*
Knowledge												
Understanding as to the extent of explaining for others	20	19.0	13	17.1	7	24.1	5	4.8	3	3.9	2	6.9
Having heard but being unable to explain	62	59.0	46	60.5	16	55.2	37	35.2	28	36.8	9	31.0
Not knowing at all	23	21.9	17	22.4	6	20.7	63	60.0	45	59.2	18	62.1
Utilization												
Under utilization	2	2.0	1	1.4	1	3.4	0	0.0	0	0.0	0	0.0
Under consideration to use	12	11.8	7	9.6	5	17.2	2	2.0	0	0.0	2	7.1
Never been considered for use	88	86.3	65	89.0	23	79.3	99	98.0	73	100.0	26	92.9

Note. Adult guardianship: knowledge, *n* = 105; utilization, *n* = 102. Civil trust system: knowledge, *n* = 105; utilization, *n* = 101.

## Data Availability

The data are available on request from the corresponding author.

## References

[1] Takase Y., Sakakibara M., Igarashi A., Kumagai Y., Nagumo A., Ogiwara M., Orimo T., Aoki N., Kudoh C. (2016). Simplified Questionnaire for Early Detection of Dementia. J. Gen. Fam. Med..

[2] Boyle P.A., Yu L., Buchman A.S., Bennett D.A. (2012). Risk Aversion Is Associated with Decision Making among Community-Based Older Persons. Front. Psychol..

[3] James B.D., Boyle P.A., Yu L., Han S.D., Bennett D.A. (2015). Cognitive Decline Is Associated with Risk Aversion and Temporal Discounting in Older Adults without Dementia. PLoS ONE.

[4] Christelis D., Jappelli T., Padula M. (2010). Cognitive Abilities and Portfolio Choice. Eur. Econ. Rev..

[5] Kim E.J., Hanna S.D., Chatterjee S., Lindamood S. (2012). Who Among the Elderly Owns Stocks? The Role of Cognitive Ability and Bequest Motive. J. Fam. Econ. Issues.

[6] Oba H., Kadoya Y., Matsuoka T., Narumoto J. (2020). Cognitive Decline Reduces Household Spending among Older People. Psychogeriatrics.

[7] Alzheimer’s Society (2013). Dementia-Friendly Financial Services.

[8] Tsoh J., Peisah C., Narumoto J., Wongpakaran N., Wongpakaran T., O’Neill N., Jiang T., Ogano S., Mimura M., Kato Y. (2015). Comparisons of Guardianship Laws and Surrogate Decision-Making Practices in China, Japan, Thailand and Australia: A Review by the Asia Consortium, International Psychogeriatric Association (IPA) Capacity Taskforce. Int. Psychogeriatrics.

[9] Statistics Bureau of Japan Population and Households of Japan 2015: Chapter 9 Population Aged 65 and Over. http://www.stat.go.jp/english/data/kokusei/2015/poj/mokuji.html.

[10] Kadoya Y., Khan M.S.R., Oba H., Narumoto J. (2020). Factors Affecting Knowledge about the Adult Guardianship and Civil Trust Systems: Evidence from Japan. J. Women Aging.

[11] Kawano Y., Yasuda A., Kinoshita T., Inaba Y., Kawashima N., Takakuwa M., Naraoka M., Narabayashi Y., Nishimura C., Hirai S. (2010). A Study of the Economic Opportunity Cost of People with Dementia of the Alzheimer Type and Their Family Carers in Japan. Jpn. J. Geriatr. Psychitry.

[12] Statistics Bureau of Japan Family Income and Expenditure Survey: 2018 Yearly Average Survey Results, Table 9. https://www.stat.go.jp/english/data/sousetai/es18.html.

[13] United Nations (2017). World Population Prospects The 2017 Revision Key Findings and Advance Tables.

[14] McKhann G., Drachman D., Folstein M., Katzman R. (1984). Clinical Diagnosis of Alzheimer’s Disease: Report of the NINCDS-ADRDA Work Group under the Auspices of Department of Health and Human Services Task Force on Alzheimer’s Disease. Neurology.

[15] Lawton M.P., Brody E.M. (1969). Assessment of Older People: Self-Maintaining and Instrumental Activities of Daily Living. Gerontologist.

[16] Koyano W., Shibata H., Nakazato K., Haga H., Suyama Y. (1991). Measurement of Competence: Reliability and Validity of the TMIG Index of Competence. Arch. Gerontol. Geriatr..

[17] Folstein M.F., Folstein S.E., McHugh P.R. (1975). “Mini-Mental State”: A Practical Method for Grading the Cogntiive State for the Clinician. J. Psychiatr. Res..

[18] Reisberg B., Ferris S.H., Anand R., Leon M.J.D.E., Schneck M.K., Buttinger C., Borenstein J. (1984). Functional Staging of Dementia of the Alzheimer’s Type. Ann. N. Y. Acad. Sci..

[19] R Core Team (2016). A Language and Environment for Statistical Computing.

[20] Scrutton J., Brancati C.U. (2016). Dementia and Comorbidities Ensuring Parity of Care.

[21] James B.D., Boyle P.A., Bennett J.S., Bennett D.A. (2012). The Impact of Health and Financial Literacy on Decision Making in Community-Based Older Adults. Gerontology.

[22] Pillemer K., Burnes D., Riffin C., Lachs M.S. (2016). Elder Abuse: Global Situation, Risk Factors, and Prevention Strategies. Gerontologist.

[23] Age UK (2015). Only the Tip of the Iceberg: Fraud against Older People.

[24] Majer R., Simon V., Csiba L., Kardos L., Frecska E., Hortobagyi T. (2019). Behavioural and Psychological Symptoms in Neurocognitive Disorders: Specific Patterns in Dementia Subtypes. Open Med..

[25] Terum T.M., Testad I., Rongve A., Aarsland D., Svendsboe E., Andersen J.R. (2019). The Association between Specific Neuropsychiatric Disturbances in People with Alzheimer’s Disease and Dementia with Lewy Bodies and Carer Distress. Int. J. Geriatr. Psychiatry.

